# Proteinases in Excretory-Secretory Products of *Toxocara canis* Second-Stage Larvae: Zymography and Modeling Insights

**DOI:** 10.1155/2014/418708

**Published:** 2014-08-14

**Authors:** Gonzalo Ernesto González-Páez, Fernando Alba-Hurtado, Carlos Gerardo García-Tovar, Raúl Argüello-García

**Affiliations:** ^1^Departamento de Ciencias Biológicas y Programa de Posgrado en Microbiología, Facultad de Estudios Superiores Cuautitlán, Universidad Nacional Autónoma de México, 54714 Cuautitlán, MEX, Mexico; ^2^Departamento de Genética y Biología Molecular, Centro de Investigación y de Estudios Avanzados-IPN, 07360 Mexico City, DF, Mexico

## Abstract

Components released in excretory-secretory products of *Toxocara canis* larvae (TES) include phosphatidylethanolamine-binding proteins (TES26), mucins (TES120, MUC2-5), and C-type lectins (TES32, TES70) and their biochemical, immunological, and diagnostic properties have been extensively studied albeit proteinase activities towards physiological substrates are almost unknown. Proteolytic activities in TES samples were first analyzed by gel electrophoresis with gelatin as substrate. Major activities of ~400, 120, and 32 kDa in TES were relatively similar over a broad pH range (5.5–9.0) and all these were of the serine-type as leupeptin abolished gelatinolysis. Further, the ~400 kDa component degraded all physiological substrates tested (laminin, fibronectin, albumin, and goat IgG) and the 120 kDa component degraded albumin and goat IgG while proteinases of lower MW (45, 32, and 26 kDa) only degraded laminin and fibronectin, preferentially at alkaline pH (9.0). By protein modeling approaches using the known sequences of TES components, only TES26 and MUC4 displayed folding patterns significantly related to reference serine proteinases. These data suggest that most of serine proteinase activities secreted *in vitro* by infective larvae of *T. canis* have intriguing nature but otherwise help the parasite to affect multiple components of somatic organs and bodily fluids within the infected host.

## 1. Introduction

Toxocariasis is caused in dog (definitive) and human (paratenic) hosts by infection with the larvae of the ascarid worm* Toxocara canis*. Puppies infected at the intestine with the adult stage of* T. canis *shed in feces large number of infective eggs into the environment. When these are accidentally ingested by a patient, the infective larva (L2) hatches, penetrates the intestinal wall, and through the circulatory system targets various organs [1]. In dogs, the prevalence of* T. canis *ranged from 5.5% to 64.7% [2–4]. In humans, recent data show a widespread prevalence of toxocariasis, which is probably the most prevalent helminthiasis in industrialized countries although a neglected and underestimated health problem [5–7]. It is clear that the hatching of infective/juvenile larvae from embryonated eggs and the migration of second- or third-stage larvae (L2-L3) through host tissues are critical steps triggering infection and pathogenesis by* T. canis*. For the hatching process, we have reported the presence of three bands of 91, 68, and 38 kDa with aspartic-type proteinase and acidic (pH < 5) activities that were induced and released into the egg perivitelline fluid (EPF) [8]. For the migratory phase, pathologies in human toxocariasis are due to* larva migrans *syndromes observed in overt (visceral and ocular) or covert (gut-, lung-, or brain-targeted) presentations [9–13]. Larvae migrating throughout bodily fluids (i.e. blood) and somatic organs shed large amounts of lipids and immunogenic glycoproteins known together as* Toxocara *excretory-secretory products (TES) which have been proposed to serve a strategy to escape the immune attack of the host [14]. Nevertheless the* larva migrans *status in toxocariasis is associated with worms lacking anchoring apparatus (oral appendages, stylets, hooks, or scolex); hence other biological functions have to be considered for TES in the completion of* Toxocara *life cycle.

Excretory-secretory products from migratory larvae of nematodes contain several kinds of components. Besides their likely active role in hatching, proteinases are considered a decisive factor for tissue invasion and extracellular digestion of host proteins [15]. In spite of the extensive studies on the reliability of using TES for immunological/molecular diagnosis and treatment assessment strategies for toxocariasis [16–19], TES components are still being characterized at further detail. Major TES components include mucins (TES120/MUC1, MUC2–5), phosphatidylethanolamine-binding proteins (TES26), and C-type lectins (TES32, TES 70) of which nucleotide and amino acid sequences have been determined by expression sequence tag and DNA cloning strategies from* T. canis* genome [20–25]. In this context, in a classical study by Robertson et al. [26], the release of two serine proteinase activities with MW of 120 and 32 kDa in TES from L2s under* in vitro *culture that displayed higher activity at pH 9 as assessed by acrylamide-gelatin gels and radio gelatin microplate assays was reported. Total enzyme activity in TES was shown to degrade elastins, collagens, and glycoproteins contained in an extracellular matrix of a rat smooth muscle cell line. It is thus conceivable not only that different kinds of proteinase activities are developmentally secreted by hatching and migratory L2s as they encounter distinct microenvironments within the infected host but also that individual proteinases have preferential activities towards distinct physiological substrates, for example, immunoglobulins and carrier proteins (albumin) in bodily fluids and fibronectin and elastin in extracellular matrix of somatic organs.

In this work we identified by gel electrophoresis the proteinases released into culture medium by* T. canis *larvae (TES) and analyzed their (physiological) substrate- and pH-dependent activities.

## 2. Materials and Methods

### 2.1. Parasite Collection and TES Preparation

*T. canis*-infected puppies were euthanized and adult worms were collected from intestines, washed with sterile saline (SS), and stored at 4°C. Egg collection from female uteri, induction of hatching, and larvae recovery were carried out as previously described [8]. To obtain TES, 10,000 larvae/mL of RPMI 1640 medium (supplemented with 1% [w/v] glucose, 100 *μ*g/mL HEPES, and 100 IU/mL penicillin-streptomycin) were incubated in 40 mL culture bottles at 37°C in a humidified atmosphere containing 5% (v/v) CO_2_. Supernatants were collected at four weekly intervals, replacing the sampled medium each time. TES samples were filtered with a Millex filter unit (0.2 *μ*m pore size), concentrated using an ultrafiltration device (Amicon), and stored at −20°C until needed. The protein concentration in these preparations was determined by a micro-Bradford technique [27].

### 2.2. Gelatin and Substrate Gel Electrophoresis

Electrophoretic analysis of proteolytic activity contained in TES was carried out as described elsewhere [8] in SDS-10% (w/v) polyacrylamide slab gels containing 0.1% (w/v) gelatin in a Mini-Protean II Electrophoresis Unit (BioRad). Other substrates used were 0.5 mg/gel laminin, 0.5 mg/gel fibronectin, 0.1% (w/v) bovine albumin, and 0.1% (w/v) goat IgG. Samples (10 *μ*g/well) were loaded in Laemmli's buffer devoid of reducing agents (dithiothreitol or *β*-mercaptoethanol) and electrophoresis was run for 90 min at 140 V at 4°C. To develop and visualize proteolytic activity, gels were then incubated in a 0.25% (v/v) Triton X-100 solution for 1 h at room temperature (RT), soaked 3 times in distilled water for 5 min each, and transferred to acetate buffer (pH 5.5), phosphate buffer (pH 7.6), or glycine buffer (pH 9.0). After further incubation for 18–20 h at 37°C in buffer, gels were stained with Coomassie blue (0.1% [w/v] in 40% [v/v] methanol-10% [v/v] acetic acid solution). Molecular weight determination was done with reference to prestained markers (BioRad).

### 2.3. Determination of Proteinase Types in TES

Samples of TES were electrophoretically separated as mentioned and gels were preincubated for 30 min at RT with one of the following compounds: 2 *μ*M* trans*-epoxysuccinyl-L-leucylamido(4-guanidino)-butane 1 (E64) to identify cysteine proteinases; 0.1 *μ*M leupeptin for serine proteinases; 0.1 *μ*M pepstatin A for aspartic proteinases; and 1 mM ethylenediaminetetraacetic acid (EDTA) for metalloproteinases. Then the proteolytic activity was developed and visualised as described above. In some cases, TES samples were separated in SDS-10% acrylamide gels without substrate and protein bands were visualized with Coomassie blue followed by destaining in the same solution lacking the dye.

### 2.4. Densitometry Analyses

Images of TES samples separated and developed by substrate gel electrophoresis were captured in Tag Image File Format (TIFF). Lanes displaying representative bands of proteolytic activity were manually analyzed using image analyzer software (Kodak Digital Science 1D v 3.0.0). In these, each band with noticeable proteolytic activity was delimited for individual determination of density and surrounding background zones were analyzed by single determinations of density. Data were collected as arbitrary density units and represented in graphics using Microsoft Excel 2010 software.

### 2.5. Protein Modeling

Amino acid sequences from* T. canis* components of TES were retrieved from GenBank or otherwise indicated and included Tc120/MUC1 (AAB05820.1), TcMUC2 (AAD49339.1), TcMUC3 (AAD49340.1), TcMUC4 (AAD49341.1), TcMUC5 (AAD49342.1), TcTES26 (UniProtKB/Swiss-Prot: P54190.1), TcTES32 (AAB96779.1), and TcTES70 (AAD31000.1). Sequences in FASTA format were used to raise protein structure models using the I-Tasser server [28, 29] that performs structural alignments between query sequences and known templates in the protein databank (PDB) library. This platform retrieves specific parameters for constructed models as the TM score (range 0-1), an index reflecting the accuracy of alignment for two given structures, and the root-mean-square-deviation (RMSD) score that indicates a measure of the differences between values predicted by retrieved models and the values actually observed in PDB templates. As recommended, we only considered significant structure alignments when TM > 0.5. The Cscore (range −5 to 2) is an index that ponderates TM and RMSD scores and allows ranking of the degrees of similarity between two given protein structures.

### 2.6. Ethics Statement

Experiments performed in this study are evaluated by official Mexican regulations (NOM-062-ZOO-1999) according to recommendations in the Guide for the Care and Use of Laboratory Animals of the National Institutes of Health, USA. The study was approved by the Internal Committee for the Care of Experimental Animals of the Postgraduate Program of Animal Production and Health (UNAM, Mexico). Adult* T. canis *worms were obtained from necropsies of dogs humanely sacrificed using an overdose of sodium pentobarbital in the canine control centers of Mexico City.

## 3. Results

**Figure 1:**
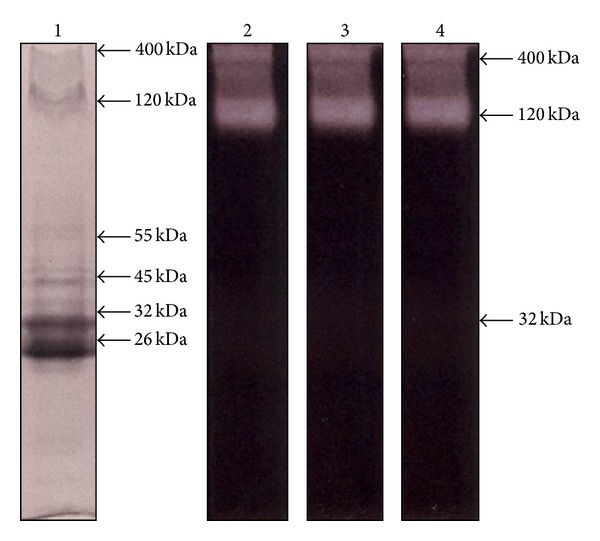
Electrophoretic detection of bands with proteolytic activity in TES of* T. canis* at different pH values.Samples of TES were loaded at a quantity of 10 *μ*g/well in slab gels of 10% (w/v) acrylamide copolymerized with 0.1% (w/v) gelatin. After electrophoretic separation, proteinase activity was developed using acetate buffer (pH 5.5,* lane 2*), phosphate buffer (pH 7.6,* lane 3*), or glycine buffer (pH 9.0,* lane 4*) and then gels were stained with Coomassie blue. In other assays, electrophoresis of TES was performed in 10% (w/v) acrylamide gels and protein bands were directly stained with Coomassie blue (*lane 1*). Molecular weight of bands with gelatinolytic activity is indicated* at the right* of the corresponding lanes.

**Figure 2:**
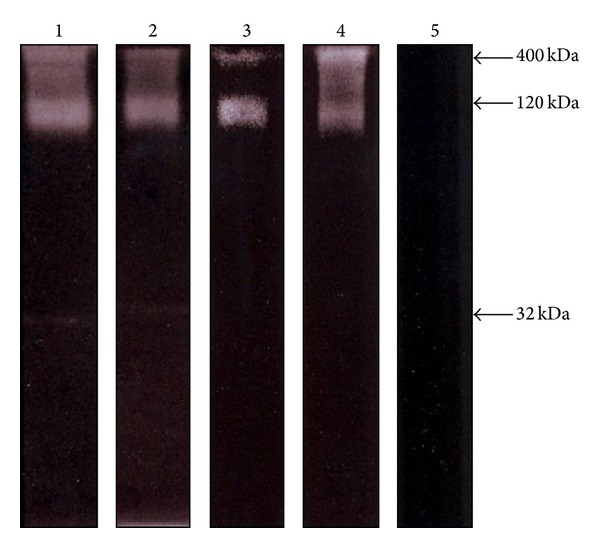
Proteolytic activity of TES components of* T. canis* analyzed by acrylamide-gelatin gel electrophoresis. Effect of proteinase inhibitors. TES components were preincubated for 30 min at RT with one of the following compounds: control without inhibitor (*lane *1); 2 *μ*M* trans*-epoxysuccinyl-L-leucylamido(4-guanidino)-butane (E64) to identify cysteine proteinases (*lane 2*); 0.1 *μ*M pepstatin A for aspartic proteinases (*lane 3*); 1 mM ethylenediaminetetraacetic acid (EDTA) for metalloproteinases (*lane 4*); and 0.1 *μ*M leupeptin for serine proteinases (*lane 5*). Samples (10 *μ*g/well) were electrophoretically separated in slab gels of 10% (w/v) acrylamide copolymerized with 0.1% (w/v) gelatin. Proteinase activity was developed using phosphate buffer (pH 7.6) and then gels were stained with Coomassie blue. Molecular weight of bands with activity is indicated* at the right*.

TES samples collected from second-stage larva cultured in RPMI 1640 medium gave yields of protein production similar to that of 8 ng/day/larvae previously reported [30]. Initial electrophoretic analyses of TES were done in polyacrylamide gels without proteinase substrate. In these, up to eight main proteins with MW of ~400, 120 (doublet), 55, 45, 32, and 26 kDa were clearly visualized (Figure 1,* lane *1). A band of 70 kDa was much less visible. When gels were copolymerized with gelatin, proteolytic (gelatinolytic) activities developed at the three different pH values employed (5.5, 7.6, and 9.0; Figure 1,* lanes* 2–4, resp.) and only the ~400 and 120 kDa bands displayed a significant activity that was similar at the different pH values evaluated. There was a very faint activity detected at Mr of 32 kDa. In order to determine the type of proteinases contained in TES, samples were subjected to electrophoretic separation using representative substrate (gelatin) and pH 5 (7.6) conditions and proteolytic activity was developed by previous treatment of gels with proteinase inhibitors with selectivity for cysteine, serine, aspartyl, and metalloproteinases. As shown in Figure 2, gelatinolytic activity in TES was virtually unaffected by E64 and EDTA (*lanes* 1 and 2), with pepstatin A exerting a partial inhibition on the ~400 and 120 kDa bands (*lane *3). However leupeptin abolished all proteolytic activity in TES (*lane *4) suggesting the presence of serine-type proteinases in these samples.

**Figure 3:**
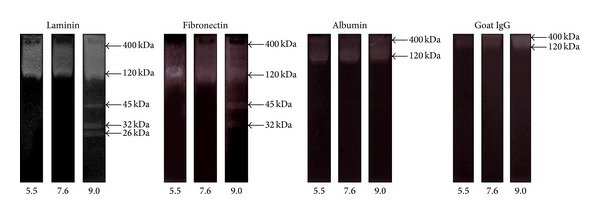
Proteolytic activity towards different substrates by TES components from* T. canis* at different pH values. TES samples (10 *μ*g/well) were electrophoretically separated in slab gels of 10% (w/v) acrylamide copolymerized with the following substrates: 0.5 mg/gel laminin, 0.5 mg/gel fibronectin, 0.1% (w/v) bovine serum albumin, and 0.1% (w/v) goat IgG. After electrophoresis, proteinase activity was developed using acetate buffer (pH 5.5), phosphate buffer (pH 7.6), or glycine buffer (pH 9.0) and then gels were stained with Coomassie blue. Molecular weight of bands with activity is indicated* at the right* of the panels indicated* at the top*. The pH values used are indicated* at the bottom* of each lane.

**Figure 4:**
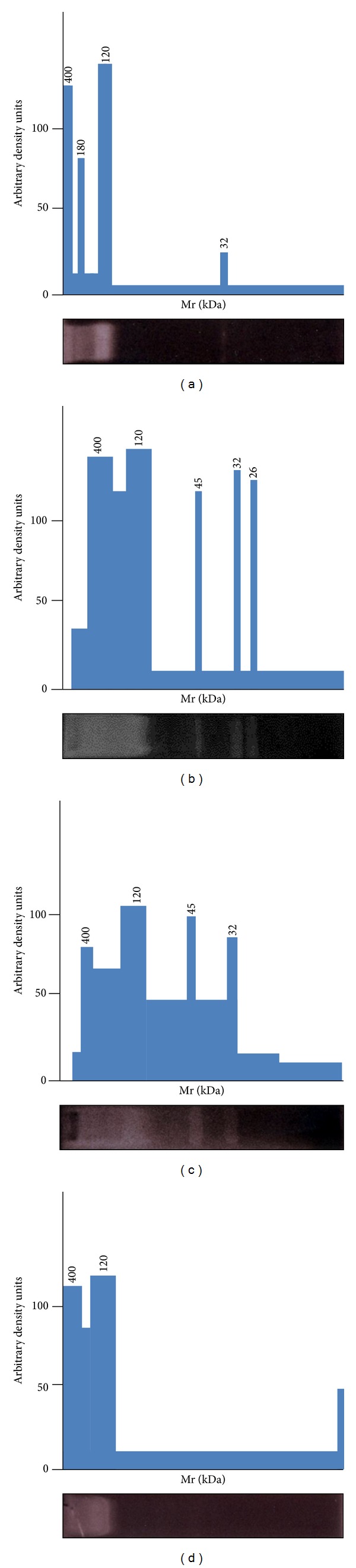
Densitometry analyses of representative proteolytic activities towards different substrates by TES components from* T. canis *L2s. TES samples previously separated by substrate gel electrophoresis were selected and densitometry analyses were carried out on tag image format file (TIFF) images delimiting each band with noticeable proteolytic activity from surrounding background zones and separate density determinations were obtained. Samples shown correspond to TES components resolved in 10% (w/v) acrylamide copolymerized with (a) 0.1% (w/v) gelatin at pH 7.6, (b) 0.5 mg/gel laminin at pH 9.0, (c) 0.5 mg/gel fibronectin at pH 9.0, and (d) 0.1% (w/v) bovine serum albumin at pH 9.0. The MW (in kDa) of each peak of density is indicated.

The presence of proteinases in TES with similar activity at an ample pH range (5.5–9.0) on gelatin, an hydrolyzed form of collagen that is a very abundant protein of host's connective tissue, prompted us to determine if these enzymes could have the same activity on a series of physiologically important proteins (i.e., albumin, IgG, fibronectin, and laminin) that second-stage larvae eventually encounter while migrating across host tissues. In these substrate gel electrophoresis assays, the bands from TES with activity towards these different substrates and at different pH values are shown in Figure 3 and summarized in Table 1. The ~400 and 120 kDa bands were similarly active towards albumin and goat IgG at all the pH values tested, except for the case of goat IgG where these two bands displayed higher activity at pH 9. Of note, bands of 400 and 120 kDa displayed a significant difficulty to be separated in gels copolymerized with these two substrates. Otherwise some distinct bands with different pH-dependent activity were observed in gels copolymerized with laminin and fibronectin. The ~400 and 120 kDa bands appeared more separated to each other and displayed activity at all pH values while bands of 45 and 32 kDa displayed proteolysis only at basic pH (9.0). Moreover a 26 kDa proteinase was detected only with laminin at pH 9.0. Because a smear in the ~400–120 kDa range was observed using laminin and fibronectin as substrates, further densitometry analyses of these smears allowed defining reliable activity bands only by the ends of the aforementioned MW range (Figures 4(b) and 4(c)). As expected, the ~400- and 120 kDa sized proteinases could be detected separately from each other by densitometry when albumin and goat IgG were tested (Figure 4(d)). In additional assays in which proteinase inhibitors were used, as expected, only leupeptin abolished proteolysis of all these four protein substrates by TES components (not shown).

In next analyses, we looked for the sequence or structural basis to explain the serine-type proteinase activities displayed by TES components. At the primary sequence level, the 8 major TES components that have been already sequenced and reported by Rick Maizels' group [20–25] show well defined domains as are the ShKT domain, also called NC6 (nematode six-cysteine) domain, SXC (six-cysteine) domain or ICR (ion channel regulator) that is present in a 2-repeat module pattern in MUC1–5 and TES26, and the C-type lectin domain (CTL) or carbohydrate-recognition domain (CRD) in TES32 and TES70. These two domains have been used as isolated sequences to determine their folding pattern using sea anemone [24] and mammalian [22] counterparts as templates, respectively. Therefore we retrieved whole-molecule models for these major TES components and searched possible serine-type proteinase homologs in PDB.

**Figure 5:**
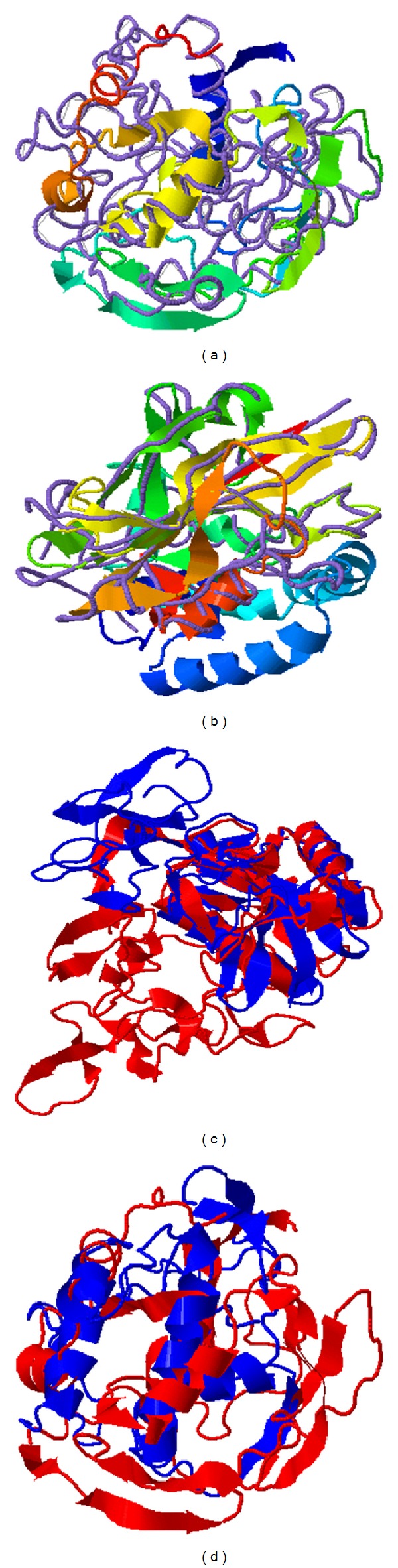
Protein modeling of* T. canis* excretory-secretory components (TES) and superimposition with homologs from other organisms ((a)-(b)) and between TES proteins with high (c) and low (d) structure homology. The following structures were superimposed using the I-Tasser platform coupled to the TM-align tool: (a) TcMUC4 (cartoon) and lipase with serine proteinase triad from* Rhizomucor miehei* (PDB 1TGL, backbone trace in purple); (b) TcTES26 (cartoon) and carboxypeptidase Y from* S. cerevisiae* (PDB 1WPX, backbone trace in purple); (c) TcTES32 (cartoon in blue) and TcTES70 (cartoon in red); (d) TES120/MUC1 (cartoon in blue) and TcMUC4 (cartoon in red).

In the case of serine proteinase activities observed at 400 and 45 kDa, none encoding sequence has been conclusively proposed. In the case of TES120/MUC1 there were no obvious homologs (TM score = 0.24, RMSD = 16.1, and Cscore = −4.52) and searches in PDB retrieved only a serine-type proteinase with low homology (human aminopeptidase A, PDB 4KxTH, Tm score = 0.443, RMSD = 5.12). For MUC2, 3, and 5 the homologs found were basically of the mucin family. Interestingly, the folding of MUC2 and MUC5 models was strikingly distinct from the remainder mucins as it was spindle-shaped while the others were globular-shaped. On the other hand, MUC4 had a reliable serine proteinase homolog which was the lipase with serine proteinase triad Ser/His/Asp from* Rhizomucor miehei* (PDB 1TGL, TM score = 0.554, RMSD = 4.90 with a sequence coverage of 0.90) (Figure 5(a)). In the primary amino acid sequence, the typical lipase serine active moiety VAVTGH**S**LGG is present as VVA**S**QAA in TcMUC4. Besides MUC4, TES26 was the only other component with a serine proteinase homolog retrieved, which was the carboxypeptidase Y from* Saccharomyces cerevisiae* (PDB 1WPX, TM score = 0.557, RMSD = 2.47 with a sequence coverage of 0.63) (Figure 5(b)). At primary sequence, the archetypal serine active moiety FHIAGESYAG is present as FNLGSPYAG in TcTES26 while the histidine-containing active moiety FTYLRVFNGG**H**MVPFDVP is apparently a divergent PSTPAANTGV**H**RYVFLVY sequence in TcTES26. Regarding TES32 and TES70, both retrieved homologs bearing the typical C-type lectin domain mediating binding to oligosaccharides; nevertheless in pair-matching comparison of protein structures among these 8 TES components, TES32/TES70 had the highest structural homology (TM score = 0.605, RMSD = 3.0) (Figure 5(c)). As reference, superimposition of TES120/MUC1 to MUC4 did not give significant homology (TM score = 0.288, RMSD = 4.88) (Figure 5(d)).

## 4. Discussion

The adaptive response to host stimuli by eggshell-enclosed larva and migratory larva of* T. canis *involves developmentally regulated processes as are the secretion of enzymes needed to facilitate larval emergence (in EPF) and tissue invasion (in TES), respectively. On the basis of the previous [8, 26] and the present* in vitro *studies, it is clear that* T. canis *larvae secrete aspartic proteinases in EPF and serine proteinases in TES.

As far as major TES components are concerned, these have been largely identified as proteins bearing several kinds of carbohydrate moieties and major bands have been identified at the sequence level as mucins (TES-120 as a member of a mucin family along to MUC2–MUC5) [21, 24, 25, 31], C-type lectins (TES-32, TES-70) [22, 23], and phosphatidylethanolamine-binding protein analogues (TES-26) [20]. A high MW component of TES (TES-400) is a glycoprotein resistant to tryptic or peptic cleavage but sensitive to staphylococcal V8 proteolysis and is also detected in TES but not in L2 surface [30–32], raising the possibility that it would be an extracellular complex of other TES. Taking into account this information, it is intriguing the appearance of serine-type proteinase activity in bands with MW of 400 (glycoprotein complex), 120 (mucin complex of MUC1 to MUC3 [33]), 45 (unknown), 32 (lectin), and 26 (phosphatidylethanolamine-binding) kDa as these components have been reported to be of distinct nature and alignments and blast searches comparing these molecules with typical serine-type proteinases at the nucleotide and amino acid levels did not reveal significant similarities. Likely explanations for this issue are that a catalytically competent serine proteinase is trapped or contained within these complexes or that the macromolecular array among individual proteins in these complexes could assemble as and resemble functionally to catalytic moieties of serine-type proteinases. On the basis of the present protein modeling studies, it is conceivable that the latter possibility would occur for TES400 and TES120 complexes and that TES26 (exhibiting proteolytic activity against laminin at pH 9) and MUC4 (supposed to be scarcely secreted by larvae along to MUC5) have a serine proteinase-like folding structure and hence activity while TES32 (broadly active against gelatin and active towards laminin and fibronectin at pH 9) could have a still undefined serine-type catalytic region as other mannose-binding proteins (MASP family) have [34]. Further proteomic approaches will shed important information in these aspects.

All of the TES-n molecules aforementioned were observed in our TES preparations stained with Coomassie blue but only the ~400 and 120 kDa components along with a novel band of 180 kDa displayed serine-type activity. This is in part consistent with the 120 (doublet) and 32 kDa serine proteinases previously reported in L2 TES (Figure 2 of [26]) though in that work a significant activity in the range of >120 kDa was also detected. Accordingly TES components have shown higher proteolytic activity at pH 9.0 which correlated partially with our observations on the appearance of higher proteolytic activity by other bands (45, 32, and 26 kDa) when pH was raised to 9.0 using laminin and fibronectin as substrates but not with albumin or goat IgG. At this point, it is remarkable that the two latter substrates, contained mainly in a bodily fluid as blood, were not degraded by the low MW (<120 kDa) serine proteinases of TES while the other two extracellular matrix-associated substrates did. Remarkably the 120 kDa-associated serine proteinase activity apparently degraded substrates at the inverse pattern. The partial substrate selectivity of the 120 kDa and low MW serine proteinases contained in TES deserves future studies. In other helminthes, serine proteinases have a developmental role in the degradation of different substrates: in* Trichuris muris *two proteinases of 85 and 105 kDa have certain specificity for collagen-like substrates [35]; in the microfilaria* Onchocerca volvulus *only male worms secrete serine/metalloproteinase activities in ES products that degrade collagen type IV, fibronectin, and laminin [36] while only L3 but not the adult stage secretes a 43 kDa-sized serine elastase [37]. Likewise the proteolytic activity of the ~400 and 120 kDa TES components towards standard (gelatin) and physiological substrates (laminin, albumin, and goat IgG) over a broad pH range (5.5–9.0) extends previous observations with elastins and collagens [26] and reinforces two in-force hypotheses: firstly, the proposed relevance of TES components for larval migration and counteraction of host immune responses, and, secondly, that proteinases released by parasites often exhibit a pH profile broader than most of its mammalian counterparts in a consistent way with their explicit extracellular function (digestion, degradation, or processing of extracellular matrix proteins) within a potentially changing microenvironment. Indeed these molecules are not as redundant as mammalian ones could be [15, 38]. In addition, it remains to be determined if any proteinase(s) contained in TES might act towards hemoglobin, a major blood component, albeit hemoglobinolytic activities in helminths extracts are more easily detected using* in solution* assays than* in gel* ones and aspartic-type proteinases are more likely related to these activities [39]. However the virtually exclusive presence of serine proteinases in TES would be related to the restricted host specificity of* T. canis *(dogs and humans) as compared to other nematode organisms with lack of host specificity such as* Trichinella spiralis *where the ES products from infective larvae contain serine, aspartic, cysteine, and metalloproteinases [40].

## 5. Conclusions

Here, we describe the presence of up to five serine-type proteinases in excretory-secretory products of the second-stage larvae of* T. canis* (400, 120, 45, 32, and 26 kDa), three of them with proteolytic activity not reported before, that would contribute to the larval migratory process that implies its distribution by circulatory fluids and invasion of somatic organs affected in the different presentations of toxocariasis. The degrading activity of these enzymes on representative blood (albumin and IgG) and extracellular matrix (fibronectin and laminin) components not only extends previous observations using elastin and collagen [26] but also reveals the partial substrate selectivity of the 120 kDa complex and the low-MW proteinases in TES. Also, the availability of amino acid sequences for the most relevant TES components [20–25] allowed through protein modeling approaches obtaining important insights into the nature of the serine proteinase activities observed and providing basis for future studies. These proteinases remain by themselves attractive targets to improve strategies for control of toxocariasis including drug design, drug evaluation, diagnosis, and prophylaxis.

## Figures and Tables

**Table 1:** Summary of bands from TES with proteolytic activity in SDS-acrylamide gels copolymerized with the indicated substrate at different pH values.

Substrate	pH		
	5.5	7.5	9.0
Gelatin	400, 120, and 32 kDa	400, 120, and 32 kDa	400, 120, and 32 kDa
Laminin	400 and 120 kDa	400 and 120 kDa	400, 120, 45, 32, and 26 kDa
Fibronectin	400 and 120 kDa	400 and 120 kDa	400, 120, 45, and 32 kDa
Albumin	400 and 120 kDa	400 and 120 kDa	400 and 120 kDa
Goat IgG	400 and 120 kDa	400 and 120 kDa	400 and 120 kDa
